# Systemic orchestration of cell size throughout the body: influence of sex and rapamycin exposure in *Drosophila melanogaster*

**DOI:** 10.1098/rsbl.2022.0611

**Published:** 2023-03-22

**Authors:** Ewa Szlachcic, Anna Maria Labecka, Valeriya Privalova, Anna Sikorska, Marcin Czarnoleski

**Affiliations:** Life History Evolution Group, Institute of Environmental Sciences, Faculty of Biology, Jagiellonian University, Gronostajowa 7, 30-387 Kraków, Poland

**Keywords:** body size, cell size, *Drosophila melanogaster*, signalling pathways, target of rapamycin

## Abstract

Along with differences in life histories, metazoans have also evolved vast differences in cellularity, involving changes in the molecular pathways controlling the cell cycle. The extent to which the signalling network systemically determines cellular composition throughout the body and whether tissue cellularity is organized locally to match tissue-specific functions are unclear. We cultured genetic lines of *Drosophila melanogaster* on food with and without rapamycin to manipulate the activity of target of rapamycin (TOR)/insulin pathways and evaluate cell-size changes in five types of adult cells: wing and leg epidermal cells, ommatidial cells, indirect flight muscle cells and Malpighian tubule epithelial cells. Rapamycin blocks TOR multiprotein complex 1, reducing cell growth, but this effect has been studied in single cell types. As adults, rapamycin-treated flies had smaller bodies and consistently smaller cells in all tissues. Regardless, females eclosed with larger bodies and larger cells in all tissues than males. Thus, differences in TOR activity and sex were associated with the orchestration of cell size throughout the body, leading to differences in body size. We postulate that the activity of TOR/insulin pathways and their effects on cellularity should be considered when investigating the origin of ecological and evolutionary patterns in life histories.

## 1. Introduction

Within modern vertebrates, the fish *Schindleria brevipinguis* matures with a body mass approximately 284 million-fold lighter than that of the blue whale *Balaenoptera musculus* [1,2]. Apparently, along with differences in body plans and life histories, organisms have evolved enormous differences in body size, which differentiate phylogenetic branches, populations [3–6] and sexes [7,8]. Moreover, changes in adult size are also commonly involved in the plasticity of organisms’ developmental responses to environmental conditions [9–12]. Such high variability in the size of organisms has long inspired scientific debate [13–16], mainly because the size achieved at maturity is a fundamental determinant of Darwinian fitness [17,18], with far-reaching consequences for physiological and ecological processes, e.g. the energy budget, mortality, competition and niche breadth [13,17]. Mechanistically, this variability involves changes in cell numbers and size as well as the amount of extracellular components, but we usually do not know the role of each of these mechanisms or their fitness consequences [8,13,18]. Emerging evidence suggests that cell size does not remain constant, changing both with the developmental environment [12,19–23] and over evolution, differentiating populations and species [19,24]. There is also direct evidence that changes in cell size are a component of plasticity- and evolutionary-related changes in adult size [7,19,21,25–28]. Undoubtedly, the number and size of the cells that make up an organism have consequences for organismal performance [29–33], but it is unclear whether the cellular structure of tissues is systemically organized throughout the metazoan body or whether it is arranged locally to suit tissue-specific functions. A clear answer to this fundamental question is unavailable largely because previous studies have rarely focused on cell-size variance among organisms and even then have tended to target single cell types [10,21,34–37]. The systemic orchestration of cell size throughout the body may be an evolutionarily conserved characteristic [38], which is partially supported by studies of invertebrates [8,12,20,39,40], vertebrates [24,41] and plants [42].

Research is only just beginning to understand how cells sense and regulate their size, with studies focusing on cell-cycle checkpoints and molecular pathways involved in cell-autonomous and systemic control of cell size [33,43]. On a macroevolutionary timescale, the evolution of cell-cycle control appears to have involved the gradual incorporation of new signalling pathways into the conserved backbone formed by AMP-activated protein kinase (AMPK) and target of rapamycin (TOR), two protein kinases that act as signalling hubs to integrate and exchange information with the entire network of regulatory pathways [44,45]. The emergence of eukaryotes probably occurred after the origin of the TOR pathway, which subsequently became regulated by the insulin pathway with the emergence of animals [44,45]. Thus, there seems sufficient evidence to consider the TOR/insulin pathways as good candidates for the primary regulatory mechanism ensuring systemic orchestration of cell size throughout the animal body. To explore this possibility, we experimentally manipulated TOR activity in fruit flies (*Drosophila melanogaster*) by rearing larvae with or without rapamycin and measuring their body size as well as the cell sizes in five organs of the eclosed adult flies. At the molecular level, rapamycin targets TOR, downregulating the activity of TOR multiprotein complex 1 [46]. Rapamycin is a bacterial antibiotic used as an immunosuppressive drug [47], with recognized anti-ageing potential [48]. Rapamycin administration to *D. melanogaster* larvae has been shown to delay development and lead to smaller adult flies and smaller cells [29,49,50], but it remains unclear how, if at all, modulation of TOR activity orchestrates cell sizes in different tissues and organs in the body. Additionally, such systemic control may not occur, as emerging (but still fragmentary) evidence in snails [51], geckos [25] and woodlice [39] indicates irregularities in cell-size changes in different tissues. Importantly, previous studies on the effects of rapamycin in flies have typically focused on larvae directly exposed to rapamycin and examined only single cell types (but see [52,53]). Clearly, we are far from possessing a satisfactory understanding of the systemic control of cell size and the involvement of the TOR signalling pathway in this phenomenon.

## 2. Methods

We used 14 genetic isolines from a wild population of *D. melanogaster* established in 2017 (49°58′00.8″N, 20°29′54.1″E) and maintained at the Institute of Environmental Sciences (Jagiellonian University, Kraków, Poland) as a source of viable random genotypes for our experimental studies [29]. Flies were maintained in polyurethane vials with foam plugs and cornmeal yeast medium (Bloomington Drosophila Stock Center, Bloomington, IN, USA). We used 40 ml vials (10 ml of food) for the stock population and 68 ml vials (20 ml of food) for the study population. Flies were kept in thermal cabinets (POL-EKO, Wodzisław Śląski, Poland) at 20.5°C, 70% relative humidity and a 12 h : 12 h L : D photoperiod. Transfers prevented generational overlap.

**Figure 1.**
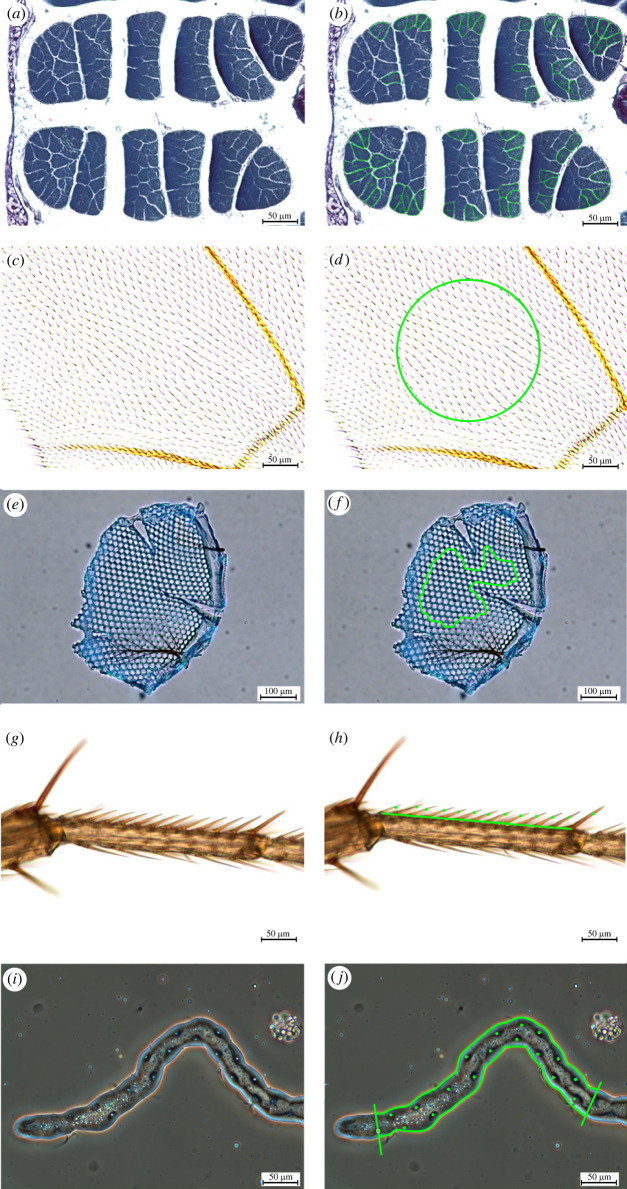
Cell-size measurements in *Drosophila melanogaster* adults. (*a*,*b*) Thorax: indirect flight muscles (mean cross-sectional area of fibres); (*c*,*d*) wings: epidermal cells (from trichome number per area unit); (*e*,*f*) ommatidia: ommatidial cells (mean area of ommatidia); (*g*,*h*) legs: epidermal cells (from bristle number per length unit); (*i*,*j*) Malpighian tubules: epithelial cells (from nuclei/nucleoli number per area unit). (*a*,*c*,*e*,*g*,*i*) show raw images, (*b*,*d*,*f*,*h*,*j*) show images with measurements. See electronic supplementary material for methodology.

Following our previous approach [29], we produced two consecutive generations under conditions of controlled larval density. Upon each transfer, we placed 10 females and five males from each isoline in a vial for 48 h for oviposition. On the second transfer, representatives of each isoline were assigned to two treatments, with oviposition performed in vials with rapamycin-supplemented food or standard food. For rapamycin treatment, we dissolved rapamycin (Alfa Aesar by Thermo Fisher Scientific, Kandel, Germany) in 96% ethanol (Linegal Chemicals, Warszawa, Poland) and added it to standard food at a 1 µM concentration. For the control, the same amount of ethanol was added to the standard food. Flies from the second generation, 1–16 days after eclosion, were anaesthetized with CO_2_ and dissected (figure 1 and electronic supplementary material for histological methodology). For each fly (112 per treatment), we measured the distance from the neck edge to the tip of the scutellum (thorax length, mm). We took a left wing, left middle leg and head from two males and two females per isoline (56 flies per treatment); in males, we also dissected the Malpighian tubules. From another two males and two females per isoline (56 flies per treatment), we obtained thoraxes. The legs (ethanol), wings (freezing), heads (methanol) and thoraxes (reagents) were preserved as indicated for further steps; Malpighian tubules were imaged immediately without fixing. We measured (figure 1) the size of epidermal cells in the wing (µm^2^) and leg (µm), ommatidial cells (µm^2^), Malpighian tubule epithelial cells (µm^2^) and dorsal longitudinal indirect flight muscle cells (μm^2^). For consistency, linear measures of leg cells were squared (μm^2^).

Thorax lengths and cell sizes were analysed with general linear mixed models (GLMMs) in R software (v.4.0.3) [54] with the lme4 [55], lmerTest [56] and car [57] packages. Figures were generated with the ggplot2 [58] and emmeans [59] packages as well as Inkscape [60]. To meet the assumptions of parametric methods, thorax lengths were cube transformed (mm^3^). The models included treatment (rapamycin versus control), sex (Malpighian tubules were obtained for males only) and the treatment × sex interaction as fixed factors and isoline as a random factor. Non-significant interactions were removed from the final models, following confirmation of models' improvements using the Akaike information criterion. To better understand independent effects of body size, sex and treatment, we analysed additional GLMMs for cell sizes with thorax length (mm) as a covariate.

## 3. Results

**Figure 2.**
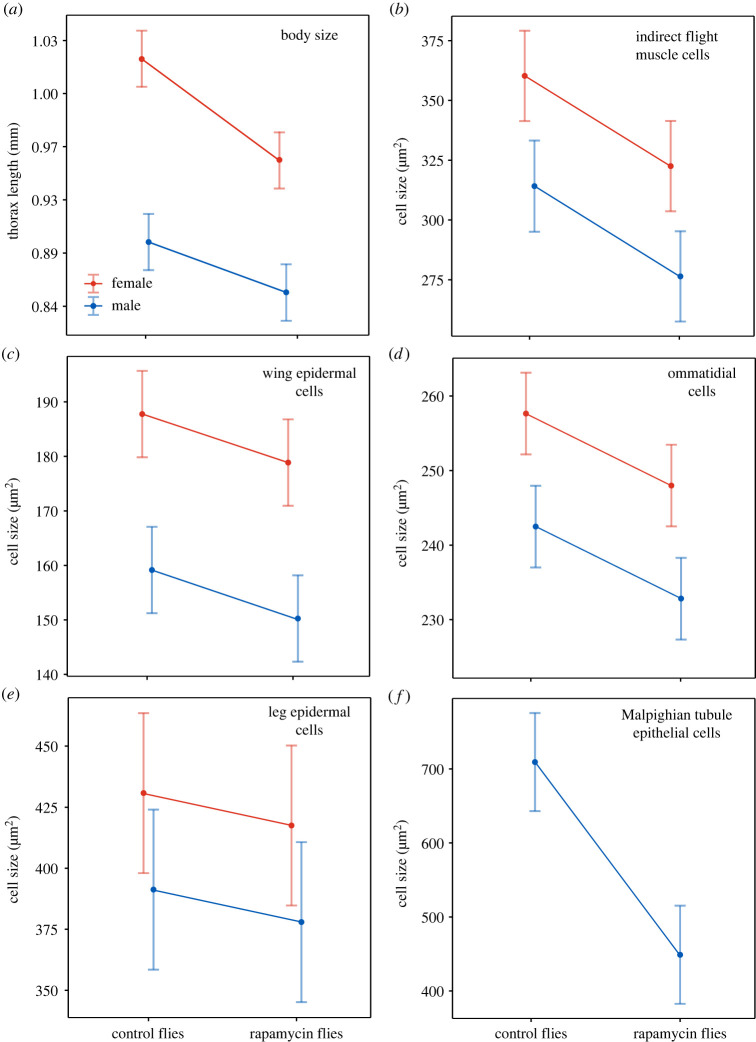
After exposure to rapamycin during development, eclosed *Drosophila melanogaster* had smaller thoraxes (*a*) and cell sizes in five tissues ((*b–f*); figure 1). Modelled means with 95% confidence intervals (table 1). Thorax length was back-transformed for display.

The interaction between treatment and sex was non-significant in all GLMMs for cells, but it was significant in the GLMM for body size (table 1). GLMMs demonstrated that rapamycin supplementation delayed development (by 12%, electronic supplementary material, table S1) and produced flies with smaller thoraxes (by 4.6% in males and by 6.4% in females; thorax length was back-transformed for calculations) and smaller cells in all cell types (table 1 and figure 2): flight muscle cells (by 12.0% in males and 10.5% in females), wing epidermal cells (by 5.6% in males and 4.7% in females), ommatidial cells (by 5.3% in males and 5.0% in females), leg epidermal cells (by 3.4% in males and 3.1% in females) and Malpighian tubule epithelial cells (by 36.7% in males). For cells in the legs, the results were not significant at *p* = 0.05 but showed a pattern consistent with other tissues. Overall, these analyses yielded nine (sex, organs) estimates of responses to rapamycin, all with consistent directions, but two (legs in males and females) showed low statistical significance. Integrating this information, we performed an *ad hoc* sign test, showing that obtaining such response consistency by chance had a very low probability (0.002). Consequently, we concluded that rapamycin induced a reduction in cell size throughout the organism, but the magnitude of this effect varied between organs.

**Table 1.** GLMM comparisons of adult *Drosophila melanogaster* after feeding diets with and without rapamycin. n.s., non-significant interactions removed from final models.

*fixed effects*	body size: thorax length (mm^3^) *N* = 224		cell size: thorax muscle (µm^2^) *N* = 111		cell size: wings (µm^2^) *N* = 112		cell size: ommatidia (µm^2^) *N* = 109		cell size: legs (µm^2^) *N* = 112		cell size: Malpighian tubules (µm^2^) *N* = 56	
	*t*	*p*	*t*	*p*	*t*	*p*	*t*	*p*	*t*	*p*	*t*	*p*
intercept (control female)	40.7	<0.0001	38.3	<0.0001	50.0	<0.0001	97.7	<0.0001	27.2	<0.0001	21.7	<0.0001
treatment (rapamycin)	−9.8	<0.0001	−3.9	0.0002	−4.7	<0.0001	−7.3	<0.0001	−1.4	0.1680	−5.9	<0.0001
sex (male)	−17.7	<0.0001	−4.8	<0.0001	−15.1	<0.0001	−8.6	<0.0001	−4.1	<0.0001		
treatment × sex (rapamycin male)	3.5	0.0006	n.s.		n.s.		n.s.		n.s.			
*random effects* variance estimates												
isoline (intercept)	0.0069		277.2		159.9		64.5		2561		1216	
residual	0.0106		2548.9		100.6		84.5		2555		27490	

GLMMs demonstrated that compared to males, females had larger thoraxes (by 13.9% in control flies and 11.8% in rapamycin flies; thorax length was back-transformed for calculations) and larger cells in all cell types (table 1 and figure 2): flight muscle cells (by 14.7% in control flies and 16.7% in rapamycin flies), wing epidermal cells (by 18.0% in control flies and 19.0% in rapamycin flies), ommatidial cells (by 6.3% in control flies and 6.6% in rapamycin flies) and leg epidermal cells (by 10.1% in control flies and 10.5% in rapamycin flies). With thorax length as a covariate (electronic supplementary material, table S2), GLMMs for cell size showed that the effect of sex disappeared for the flight muscles and legs, and the effect of treatment disappeared for the wings and legs.

## 4. Discussion

Rapamycin supplementation of *D. melanogaster* larvae reduced cell sizes in all five organs assessed in adult flies, with females consistently exhibiting larger cells than males; this finding indicates strong systemic orchestration of cell sizes throughout the body. Specifically, depending on the organ and sex of an individual, rapamycin induced a 3.1–36.7% reduction in cell size; females had cells 6.3–19.0% larger than those of males. The systemic cell-size changes were involved in the origin of body-size differences, such that smaller adult flies (rapamycin/male individuals) had smaller cells in all organs than the larger adult flies (control/female individuals). Consistently, when we analysed our cell-size data while controlling for body size, all organs showed a positive relationship between cell size and individual differences in thorax length, and the independent effects of treatment and sex even disappeared in some organs. Coupling between body size and cell size has been reported in previous studies [7,24,26,28,51,61], although this evidence was largely based on the measurements of single cell types (but see [8]). Systemic coordination of cell-size changes has been suggested by some interspecies [8,12,24,38,42] and intraspecies [20,39–41] comparisons, but until now, never demonstrated experimentally by manipulating the activity of cell-cycle regulatory pathways. Notably, while our results strongly support the role of systemic orchestration of cell size throughout the body, the magnitude of rapamycin effects appeared to vary between organs. We can only speculate that this response variance indicates some degree of tissue autonomy in cell-size regulation. In fact, emerging evidence of the involvement of cell-cycle control in sex determination in *D. melanogaster* suggests that this control system includes autonomous regulation of cell size, which in females, but not in males, is additionally associated with a systemic signalling network via TOR/insulin pathways [43,62,63]. In the light of our results, this sex-dependent regulatory system is tolerant to systemic changes in TOR pathway activity, maintaining sex differences in the cellular composition of organisms regardless of variation in TOR signalling activity. Indeed, Rideout *et al*. [62] showed that downregulation of TOR activity in larvae alone was not sufficient to alter sexual dimorphism of body size in adult *D. melanogaster*, whereas this dimorphism was blurred when the TOR and insulin pathways were simultaneously inhibited. Importantly, previous studies of sex-determination regulatory pathways in *Drosophila* have considered only single cell types; our results further suggest that while each cell in an organism maintains its sex identity by autonomously regulating its size, collectively, these regulations lead to highly orchestrated sexual differences in the cellular structure of tissues and organs. Overall, our evidence clearly shows that systemic regulation of cell size can be achieved via modulation of TOR activity. As part of the TOR/insulin signalling network, TOR is deactivated under natural conditions by a shortage of incoming nutrients and oxygen, which promotes autophagy and slows ageing [64,65]. This suggests that TOR plays a central role as a switch of resource allocation ‘sinks', ultimately leading to the life-history strategy of an organism. In fact, *D. melanogaster* has evolved latitudinal clines in coupled changes in cell size, body size and genes affecting TOR activity [66–68], supporting the view that the activity of TOR and its phenotypic effects (cell size and body size) are not selectively neutral [13].

Taken together, our results and published data suggest that systemic orchestration of cell size and its contribution to the emergence of body-size variation occur commonly in nature, suggesting that these understudied phenomena are manifestations of adaptive responses to selection. Indeed, evidence suggests that *D. melanogaster* with rapamycin-induced reductions in the size of wing epidermal cells outperform flies with larger cells during flight in oxygen-poor conditions, suggesting a causal link between the cellularity of the body and organismal performance [29]. In support of this, we showed that changes in cell size in one cell type were associated with changes in cell size in other cell types, including flight muscles. However, it remains unclear how the collective effect of cell size in all tissues shapes organismal performance such that the synchronization of cell-size changes in different tissues confers evolutionary benefits. This is not a trivial question, especially as cell properties such as cell number, cell size, cell shape and organelle content should correspond closely to tissue-specific functions. The theory of optimal cell size (TOCS) [21,25,31,33,38,41,69,70] predicts that the cellular composition of an organism is optimized to selection pressures through a compromise between the cost of plasma-membrane maintenance and the cell capacity to perform physiological functions. The relatively large area of the plasma membrane of small cells should increase the rates of oxygen and nutrient fluxes but incurs costs imposed by ionic gradients and the need to maintain adequate membrane structure. In the TOCS framework, orchestration of the cellular composition of tissue throughout the body would maximize the benefits of having large or small cells, specifically, by providing more efficient systemic energy savings or more efficient systemic transport of oxygen and nutrients, respectively. Certainly, maximizing the performance of highly specialized physiological functions, e.g. catabolic versus anabolic processes, can require specific surface-to-volume ratios or organelle contents, which could explain the reported irregularities in cell-size changes in different tissues [41]. Such effects might also explain why we did not observe the same magnitude of response to rapamycin in all cell types.

In summary, we showed that organisms use developmental mechanisms to coordinate cell size in different organs and tissues and that this systemic cellular orchestration participates in shaping the life-history strategy. For the first time, our study demonstrates the role of the TOR pathway in this cell-size coordination, which enables synchronization of the cellular composition of different organs in the body. Importantly, our results also suggest that the developmental sex-determination pathways involve tight coordination of cell size in different tissues of each sex, despite their cell-autonomous nature, as revealed by recent studies. This phenomenon deserves further investigation, especially because sexes often show different physiologies and life histories, including differences in longevity and susceptibility to different health issues [63]. We postulate that the activity of TOR/insulin pathways, with their systemic cellular effects, should be considered more frequently as part of various ecological and evolutionary patterns, such as the temperature–size rule (TSR) in ectotherms, Bergmann's rule, Foster's rule and Cope's rule [9,71–75]. The incorporation of this activity can provide a better understanding of the origins of fundamental biological phenomena, including sexual dimorphism, phylogenetic and geographical trends in life histories and the developmental responses of ectotherms to climate change.

## Data Availability

The datasets, R code and materials supporting this article have been uploaded as part of the electronic supplementary material [76]. The early version of the manuscript was deposited at *bioR_X_iv* - the preprint server for Biology: https://doi.org/10.1101/2023.01.11.521715 [77].
